# Effectiveness of Mobile Applications for Promoting Healthy and Active Lifestyles During Pregnancy and the Postpartum Period: A Systematic Review

**DOI:** 10.3390/healthcare14193261

**Published:** 2026-10-01

**Authors:** Laura Massa Alves, Cristiana Mercê, Ana Urbano Morais, Rita Santos-Rocha

**Affiliations:** 1 ESDRM—Sport Sciences School of Rio Maior, Santarém Polytechnic University, 2040-413 Rio Maior, Portugalcristianamerce@esdrm.ipsantarem.pt (C.M.);; 2 SPRINT—Sport Physical Activity and Health Research & Innovation Center, Santarém Polytechnic University, 2040-413 Rio Maior, Portugal

**Keywords:** mHealth applications, effectiveness, functionalities, characteristics, pregnancy, postpartum

## Abstract

**Highlights:**

**What are the main findings?**

- Across 20 randomised controlled trials evaluating 18 pregnancy- and postpartum-specific mHealth applications, most interventions demonstrated improvements in at least one behavioural or clinical outcome; however, effects were more consistently observed for lifestyle behaviours than for clinical outcomes.
- Effective interventions most commonly included high-quality health education, with personalisation, self-monitoring, feedback, and interaction with healthcare professionals also frequently reported; diet and physical activity were addressed far more often than stress management and sleep.
- Information quality and clinical appropriateness were the app characteristics most consistently identified among effective interventions, whereas usability, accessibility, and especially data security and privacy were incompletely addressed or poorly reported across many apps.

**What are the implications of the main findings?**

- Perinatal mHealth apps should be designed as evidence-based, clinically appropriate tools that combine trustworthy health education with tailored self-monitoring, feedback, and meaningful access to healthcare professionals.
- Future interventions should assess both behavioural and clinical outcomes over longer follow-up periods, because changes in diet, physical activity, or self-management may occur before measurable improvements in maternal, foetal, or neonatal clinical outcomes.
- Developers and researchers should prioritise privacy, data security, usability, and equitable access, while expanding app content beyond diet and physical activity to include sleep and stress management; more standardised reporting and rigorous evaluation are needed to strengthen generalisability.

**Abstract:**

**Background**: Pregnancy and the postpartum period are unique stages in a woman’s life, characterised by profound physiological, psychological, and social changes that require continuous adaptation. During these periods, women frequently seek reliable health information to support healthy behaviours and informed decision-making. Advances in information and communication technologies have facilitated rapid and convenient access to health-related information through smartphones, tablets, and other digital devices, making mHealth applications increasingly popular as tools to support maternal health. **Objective**: This systematic review aimed to analyse the effectiveness of mHealth applications in promoting healthy and active lifestyles during pregnancy and the postpartum period. A secondary objective was to identify the key characteristics and functionalities associated with effective mHealth applications. **Methods**: A comprehensive literature search was conducted in three electronic databases (PubMed, Web of Science, and EBSCO) covering publications from 2016 to 2025. Studies were eligible if they included pregnant or postpartum women, evaluated mHealth applications designed to promote healthy and active lifestyles, and compared women using these applications with those receiving usual care, traditional information resources, or no intervention. Outcomes included improvements in physical activity, healthy eating behaviours, sleep quality, and stress management. Only registered or protocol-based randomised controlled trials (RCTs) were included. Methodological quality was assessed using the Downs and Black checklist. **Results**: Twenty RCTs met the inclusion criteria. The mean methodological quality score was 20.00 ± 1.45. Eighteen perinatal mHealth applications were identified, of which 16 demonstrated effectiveness according to the predefined clinical and/or behavioural outcomes evaluated in the respective interventions. Among the effective applications, the most frequently reported functionalities were health education, followed by personalisation, self-monitoring, feedback, and interaction with healthcare professionals. Regarding app characteristics, information quality was the most frequently identified feature, followed by clinical appropriateness, usability, accessibility, and data security and privacy. **Conclusions**: Among the applications classified as effective, high-quality health information, clinical appropriateness, data security and privacy, and functionalities such as health education, interaction with healthcare professionals, self-monitoring, personalized feedback, and personalization were frequently observed. Nevertheless, further improvements are needed in the quality of health information, data security and privacy, and the integration of communication with healthcare professionals to maximise the effectiveness of mHealth applications designed for pregnancy and the postpartum period.

## 1. Introduction and Research Question

Pregnancy and the postpartum period are unique stages in a woman’s life during which the need to seek and acquire reliable, up-to-date information is particularly high, enabling women to experience these periods with greater confidence and reassurance [1].

In recent years, there has been a substantial increase in the number of mobile applications designed for the perinatal period, with these applications becoming an increasingly common source of health information for women. However, considerable variability exists in the quality and evidence base of the information provided by these applications, as many are neither developed within clinical settings nor evaluated using validated quality assessment instruments. This variability may ultimately affect their effectiveness and safety [2,3,4].

mHealth applications may incorporate multiple functionalities aimed at supporting healthy lifestyle behaviours and facilitating behaviour change, including health education, self-monitoring, feedback, personalisation, social support, interaction with healthcare professionals, and features targeting specific lifestyle domains such as healthy eating, physical activity, sleep, and stress management [5,6,7]. In addition to their functionalities, the quality of mHealth applications may influence their effectiveness, safety, and acceptability, particularly in relation to information quality, usability, accessibility, clinical appropriateness, and data security and privacy [7,8].

Evidence from several studies highlights the potential of mHealth applications during pregnancy and the postpartum period. Perinatal applications incorporating different functionalities have been associated with improvements in health-related behaviours and maternal, foetal, and neonatal well-being [9,10,11]. Nevertheless, despite the increasing adoption of mHealth technologies during pregnancy and the postpartum period, important limitations remain regarding the quality, credibility, and consistency of the information and features provided by these digital tools [12].

Although previous studies have investigated the use of mHealth applications during the perinatal period, the evidence regarding their effectiveness in promoting healthy lifestyle behaviours remains heterogeneous, and it is unclear which application functionalities and quality characteristics are most consistently associated with effective interventions. Therefore, a systematic synthesis of the available evidence is warranted to clarify the effectiveness of these applications and identify the characteristics and functionalities associated with more effective interventions.

Accordingly, the primary objective of this systematic review was to evaluate the effectiveness of mHealth app-based interventions in promoting active and healthy lifestyles during pregnancy and the postpartum period. A secondary objective was to identify the key characteristics and functionalities required or recommended for effective mHealth applications.

## 2. Methods

### 2.1. Protocol and Registration

The protocol for this systematic review was developed in accordance with the Preferred Reporting Items for Systematic Reviews and Meta-Analyses (PRISMA) 2020 statement, which comprises a 27-item reporting checklist [13]. A preliminary search of the PROSPERO database identified no registered systematic reviews addressing the same research topic. Therefore, the protocol [14] was prospectively registered in PROSPERO under the registration number CRD420251150046 [15]. The research question was formulated using the PICOS (Population, Intervention, Comparison, Outcomes, and Study design) framework [16].

### 2.2. Eligibility Criteria

Eligibility criteria were defined according to the PICOS framework (Population, Intervention, Comparison, Outcomes, and Study design), adapted to the specific research question and the characteristics of the available evidence [17].

- P (Population): Pregnant women or women up to six months postpartum.
- I (Intervention): Use of mobile health (mHealth) applications designed to promote active and healthy lifestyles. App-based interventions incorporating additional components, such as wearable devices, behavioural support, counselling, or other digital tools, were eligible when the mobile application represented an integral component of the intervention.
- C (Comparison): Intervention studies comparing an mHealth application with usual care, no intervention, traditional sources of health information, or an alternative digital/mHealth intervention. Women receiving usual care, no intervention, traditional sources of health information, or an alternative digital/mHealth intervention.
- O (Outcomes): Effectiveness of mHealth interventions in improving lifestyle-related behaviours including improvements in healthy eating, physical activity, sleep quality, and stress management, as well as the identification of app functionalities reported as necessary or desirable. App characteristics and functionalities were extracted as secondary variables to address the secondary objective of the review and were not, by themselves, required as an eligibility outcome.
- S (Study design): Randomised controlled trials (RCTs). In addition to the study design requirement, studies were eligible only when prospective trial registration or a previously published or registered study protocol could be identified. This additional methodological criterion was predefined in the review protocol [14] and was adopted to enhance methodological transparency and reduce the potential risk of selective reporting.

Studies were excluded when they did not meet one or more of the predefined population, intervention, comparison, outcome, study design, or methodological eligibility requirements.

### 2.3. Search Strategy and Data Sources

The literature search was conducted in the electronic databases PubMed, Web of Science, and EBSCO between 23 and 25 July 2025. These databases were selected to provide complementary coverage of biomedical, multidisciplinary, and health-related literature relevant to pregnancy, postpartum care, physical activity, lifestyle behaviours, and mHealth interventions. The search strategy was prespecified in the review protocol [14] and the same Boolean search strategy was applied across the three databases: (pregnancy OR pregnant OR puerperium OR postpartum OR postnatal) AND (app OR e-health OR m-health) AND (exercise OR physical activity OR lifestyle OR nutrition OR sleep OR stress).

The strategy combined free-text terms related to the perinatal period, mHealth applications, and active and healthy lifestyle behaviours. In PubMed, the terms “pregnancy”, “exercise”, and “lifestyle” were also searched using Medical Subject Headings (MeSH), where applicable.

### 2.4. Study Selection

Study selection was performed according to the eligibility criteria described above. Records retrieved from each database were exported and screened independently by two reviewers (LMA and AUM). Screening was conducted in three stages: title screening, abstract screening, and full-text assessment. Reasons for exclusion were documented and discussed during consensus meetings. At the full-text stage, studies were excluded when they did not meet one or more of the predefined eligibility criteria, with the specific reason for exclusion recorded in the PRISMA flow diagram. The availability of prospective trial registration or a previously published or registered study protocol was also verified at the full-text stage. Any disagreements between the two reviewers were resolved by consultation with a third reviewer (CM).

Screening decisions and extracted information were recorded independently by each reviewer. The literature search was conducted between 23 and 25 July 2025.

### 2.5. Data Extraction and Operational Definitions

The following data were extracted from each included study using a standardised data extraction table:

- Author and year of publication;
- Study design;
- Methodological quality assessed using the Downs and Black Checklist (27 items);
- Sample size;
- Participants’ age;
- Intervention duration;
- Outcome assessment instruments;
- mHealth application functionalities:
  ◦ Health education;
  ◦ Self-monitoring;
  ◦ Feedback;
  ◦ Personalisation;
  ◦ Interaction with healthcare professionals;
  ◦ Social support features;
  ◦ Artificial intelligence (AI);
  ◦ Healthy eating;
  ◦ Physical activity;
  ◦ Stress management and mental health;
  ◦ Sleep.
- Application quality characteristics:
  ◦ Information quality;
  ◦ Usability and user experience;
  ◦ Data security and privacy policies;
  ◦ Clinical appropriateness for perinatal care;
  ◦ Accessibility.
- Outcomes related to changes in active and healthy lifestyle behaviours before and after the intervention.

Within the context of mHealth applications, functionalities refer to the tools and features made available to users. Common functionalities include health education, self-monitoring, and personalised feedback, which are recognised as key components for promoting behaviour change [6]. In addition, mHealth applications may incorporate social interaction features, artificial intelligence (AI), and functionalities specifically designed to promote healthy lifestyle behaviours, including healthy eating, physical activity, sleep, stress management, and mental health, all of which are essential for improving health outcomes [7].

Based on these resources, eleven functionalities were considered in the present review. For each functionality, the reviewers assessed whether the feature was explicitly described as being available within the application or intervention. A functionality was classified as present when the included study provided sufficient information to identify that feature, whereas it was classified as not reported when no such information was available. Functionalities were not inferred solely from the general purpose of the application or from the presence of related intervention outcomes. Seven were classified as general functionalities, as described by Michie et al. [5] and the World Health Organization (WHO): health education, self-monitoring, feedback, personalisation, social support, interaction with healthcare professionals, and AI. The remaining four functionalities addressed key healthy lifestyle behaviours, namely healthy eating, physical activity, sleep, and stress management [5,7].

Health education is defined as a set of strategies aimed at improving individuals’ knowledge, skills, and behaviours, enabling them to make informed decisions that promote both physical and mental health [7,18]. Self-monitoring is defined as the systematic observation and recording of an individual’s behaviour to increase awareness and facilitate behaviour change, particularly in relation to lifestyle habits. Feedback provides users with information regarding their performance in relation to a predefined goal or expected standard, whereas personalisation refers to the adaptation of interventions to users’ individual characteristics, a feature that has become increasingly common in mHealth applications [5]. Self-monitoring, feedback, and personalisation are widely recognised as core behaviour change techniques and are consistent with WHO recommendations advocating the recording of health-related data and the delivery of tailored communication according to users’ individual characteristics [5,7,18]. Feedback was considered present when the application provided information to users about their behaviour or performance, including progress towards a goal, whereas personalization required explicit adaptation of content, recommendations, goals, or communication according to individual user characteristics or data. Interaction with healthcare professionals represents another important functionality of mHealth applications, as it reinforces clinical guidance, promotes continuity of care, and enhances users’ confidence and perceived safety [19]. Likewise, social support is recognised as an effective behaviour change technique involving emotional support provided by others. Within mHealth applications, this functionality is commonly implemented through social features such as online communities, discussion forums, and chat functions that facilitate interaction among users [5]. The integration of AI into mHealth applications enables the use of algorithms capable of analysing user data and generating automated, personalised recommendations, thereby supporting adaptive interventions and tailored feedback according to individual user profiles [10]. AI was considered present when the application was explicitly described in the included publication as using AI-based or machine-learning algorithms to analyse user data, generate predictions or recommendations, or adapt intervention content or feedback. General automated functions or predefined decision rules were not classified as AI unless the publication explicitly identified them as such.

Evidence from several studies highlights the importance of mHealth applications during pregnancy and the postpartum period. Perinatal applications incorporating functionalities such as health education, self-monitoring, feedback, personalisation, and social support have been shown to facilitate the adoption of healthier lifestyles, contributing to improved maternal, foetal, and neonatal well-being [9,10,11]. Nevertheless, recent evidence reported by Alves et al. [12] indicates that despite the increasing adoption of mHealth technologies during pregnancy and the postpartum period, important limitations remain regarding the quality and credibility of the information provided, potentially compromising the safety of these digital tools. Accordingly, the authors emphasise the importance of involving healthcare professionals in the design, development, evaluation, and validation of mHealth applications to ensure that the information provided is evidence-based, up to date, and responsive to women’s healthcare needs [12].

Regarding functionalities specifically related to active and healthy lifestyles, four domains were considered: healthy eating, physical activity, sleep, and stress management. Healthy eating functionalities typically promote healthier dietary behaviours through nutritional recommendations and daily dietary self-monitoring. Similarly, physical activity functionalities based on current clinical guidelines enhance users’ health literacy and motivation to engage in regular exercise while enabling the monitoring of variables such as exercise duration, daily step count, and other physiological parameters through mobile devices, either independently or integrated with wearable technologies, thereby facilitating continuous self-monitoring [10,19,20].

Characteristics, in contrast, refer to the quality attributes of mHealth applications that influence their effectiveness, safety, and user acceptance. Beyond functionalities, application quality should be evaluated according to characteristics including information quality, usability, accessibility, clinical appropriateness, and data security and privacy, all of which are recognised as key determinants of the effectiveness of mHealth interventions [7,8].

Information quality refers to the reliability, accuracy, and currency of the information provided, which should be supported by high-quality scientific evidence, clinically appropriate for the target population, and preferably developed or reviewed by healthcare professionals [7,8].

Usability refers to the ease with which users can navigate and interact intuitively with an application, thereby facilitating user engagement in the intended tasks [8]. However, usability should not rely solely on users’ subjective perceptions. It is desirable that validated instruments specifically developed for evaluating mHealth applications, such as the mHealth App Usability Questionnaire (MAUQ), are employed [21]. Accessibility, on the other hand, concerns the extent to which an application can be easily accessed and used by diverse population groups across different socioeconomic and cultural contexts. The WHO considers free access to be an important criterion contributing to the accessibility of mHealth applications [7].

Mobile applications (mHealth) used in clinical interventions, particularly those involving the collection, storage, and transmission of sensitive health data, as commonly occurs during pregnancy and the postpartum period, raise important concerns regarding data security and privacy. These aspects are fundamental to ensuring the effectiveness, trustworthiness, and safe use of these technologies by women [22]. Within mHealth interventions, effectiveness should ideally be evaluated using both clinical and behavioural outcomes, as behaviour change constitutes a fundamental mechanism through which improvements in health outcomes are achieved [7,23].

App functionalities and characteristics were extracted from information reported in the included publications and their supplementary materials, when available; the applications themselves were not independently assessed or tested by the review team.

### 2.6. Quality Assessment

The methodological quality of the included studies was assessed using the Downs and Black Checklist, a validated instrument comprising 27 items designed to evaluate reporting quality, internal validity, external validity, and methodological rigour in intervention studies, including randomised controlled trials [24]. Quality assessment was independently performed by two reviewers, with disagreements resolved by consensus.

### 2.7. Definition of Intervention Effectiveness

Within mHealth interventions, effectiveness was evaluated using both clinical and behavioural outcomes, considering behaviour change as an important mechanism through which improvements in health outcomes may be achieved [7,23].

For the purposes of this review, an intervention was considered effective when its primary clinical or behavioural outcome was achieved. When the primary outcome was not met, the intervention was considered effective only when secondary outcomes demonstrated improvements consistent with the intervention objectives and represented clinically or behaviourally relevant effects [25].

Primary outcomes were considered the most important endpoints defined before the beginning of the study and used as the main measures for determining intervention success or failure. Secondary outcomes were considered additional endpoints providing supporting evidence for the primary outcome or further information relevant to the research question [25].

This approach was adopted because mHealth interventions may target multiple interconnected clinical and behavioural outcomes, and improvements in secondary behavioural outcomes may represent meaningful intervention effects even when the primary clinical endpoint is not achieved. In particular, behavioural outcomes may reflect changes in lifestyle behaviours that are relevant to the intended mechanism of action of mHealth interventions and may contribute to longer-term health benefits [7,23]. Therefore, secondary outcomes were considered in the effectiveness classification only when they were consistent with the intervention objectives and provided evidence of a meaningful effect within the scope of the intervention.

These criteria were applied to the outcomes reported in the included studies to determine whether each intervention demonstrated evidence of effectiveness within the scope of the respective study objectives.

Effectiveness was assessed at the individual study/intervention level, whereas app functionalities and characteristics were summarized at the application level to address the secondary objective of the review.

### 2.8. Synthesis of Results

Given the heterogeneity of the included interventions, populations, outcome measures, and reported results, a narrative and descriptive synthesis was conducted. The extracted data were first organised according to study characteristics, methodological quality, intervention effectiveness, application characteristics, and application functionalities.

For the synthesis of intervention effectiveness, findings were organised according to the type of outcomes assessed, namely clinical outcomes, behavioural outcomes, or both clinical and behavioural outcomes, based on the predefined effectiveness criteria described in Section 2.7. Studies showing no evidence of effectiveness according to these criteria were also identified.

The characteristics and functionalities of the applications were then synthesised across studies by identifying their presence, partial presence, or absence, allowing comparison of the features reported in interventions classified as effective or not effective. The results were presented descriptively in Tables 1–3 and subsequently interpreted according to the primary and secondary objectives of the review. A meta-analysis was not performed because the included studies demonstrated substantial heterogeneity in intervention characteristics, participant samples, outcome domains, assessment instruments, and reporting of results, which limited the comparability of findings and did not allow meaningful quantitative pooling across studies. Therefore, a quantitative synthesis of a global intervention effect was considered inappropriate. The synthesis instead focused on identifying patterns across studies regarding intervention effectiveness and the characteristics and functionalities of mHealth applications associated with effective interventions.

## 3. Results

### 3.1. Selection of Studies

The initial database search identified 1536 records across the three electronic databases (PubMed, EBSCO, and Web of Science). Following duplicate removal, screening, eligibility assessment, and full-text review, twenty studies met the predefined inclusion criteria and were included in the systematic review (Figure 1).

Outcomes are commonly classified as primary and secondary. The primary outcome represents the most important endpoint, is defined before the study begins, and serves as the main measure for determining the success or failure of the intervention. Secondary outcomes provide supporting evidence for the primary outcome or offer additional insights into the research question [25]. These principles were used to determine the effectiveness of the interventions included in this review, as presented in Table 1, Table 2 and Table 3.

### 3.2. Study Characteristics

All twenty studies implemented an intervention using a perinatal-specific mHealth app, with the results presented in Table 1, Table 2 and Table 3. The total number of apps was eighteen, as the studies by Kennelly et al. [26] and Ainscough et al. [27] used the PEARS app, while the studies by Chen et al. [28] and Chen et al. [29] used the MyHealthyWeight app, with distinct intervention approaches [26,27,28,29] (see Table 1 and Table 2).

More than half of the studies (*n* = 15) implemented interventions focused on clinical outcomes (e.g., number of women with excessive gestational weight gain, incidence of gestational diabetes mellitus, 2 h plasma glucose levels after the oral glucose tolerance test (OGTT) in the postpartum period, and postpartum depression), whereas the remaining five studies targeted behavioural outcomes, such as daily step count, dietary intake, physical activity, sedentary behaviour, and mental health. It should be noted that, in most studies with clinical primary outcomes, secondary outcomes were related to behaviours with the potential to promote lifestyle changes during pregnancy and the postpartum period.

**Table 1:** Results (authors, country, downs and black score, study design, objective, app name, platform, population, sample size, mean age, assessment instruments, intervention duration).

Authors | Country | D&B Score	Study Design	Objective	App Name | Platform	Population | Sample Size (*n*) | Mean Age (Years ± SD)	Assessment Instruments	Intervention Duration
Choi et al. (2016) [30]|USA | 20	Pilot RCT	Promotion of Physical Activity Among Physically Inactive Pregnant Women	**MoTHER** trial | Smartphone (iOS/Android) + Fitbit Ultra	Physically inactive pregnant women | IG (*n* = 15): 32.9 ± 2.5; CG (*n* = 15): 34.5 ± 2.5	Fitbit Ultra; Stanford Brief Physical Activity Survey; Physical Activity Stages of Change; Self-Efficacy for Physical Activity; Barriers to Being Active Quiz; Social Support and Exercise Survey; CES-D; Pregnancy Discomfort Checklist	12 weeks
Chan et al. (2019) [31]| China | 21	RCT	To evaluate the effectiveness of a smartphone-based intervention for educating expectant parents during pregnancy.	**iParent |** Smartphone (operating system not specified)	Primiparous pregnant women (<24 weeks’ gestation) | IG (*n* = 330): 31.3 ± 4.6; CG (*n* = 330): 31.2 ± 4.5. Mothers (4 weeks postpartum): IG, *n* = 218; CG, *n* = 225.	Chinese version of the EPDS; DASS; 12-Item Short Form Health Survey (SF-12); Sociodemographic Characteristics Questionnaire; App usage records (logins, duration of use).	Pregnancy to 4 weeks postpartum
Kennelly et al. (2018) [26] |Ireland | 19	RCT	To evaluate the effect of a behavioural lifestyle intervention (physical activity and diet), supported by a smartphone app, on the incidence of gestational diabetes mellitus (GDM) among women with overweight and obesity.	**PEARS |** Smartphone (operating system not specified)	Pregnant women; BMI ≥25 kg/m^2^ | IG (*n* = 278): 32.8 ± 4.6; CG (*n* = 287): 32.1 ± 4.2	Oral glucose tolerance test (OGTT); food diaries; two validated questionnaires assessing PA and lifestyle; physical activity questionnaire (METs); anthropometric assessment and body composition measured by bioelectrical impedance analysis (BIA); blood sampling; foetal biometry; collection of clinical data on labour, delivery, the postpartum period, and neonatal outcomes.	<15 to 28 weeks’ gestation
Ainscough et al. (2020) [27] | Ireland | 19	RCT	To investigate the impact of a behavioural lifestyle intervention supported by a smartphone app among pregnant women with overweight and obesity.	**PEARS |** Smartphone (operating system not specified)	Pregnant women with a BMI of 25–39.9 kg/m^2^; 10–16 weeks’ gestation | IG (*n* = 278): 32.84 ± 4.60; CG (*n* = 287): 32.22 ± 4.23	Food diaries analysed using Nutritics; PA questionnaire adapted from the SLÁN 2002 Survey; calculation of MET-min/week; Stage of Change Questionnaire; app usage data (number of logins and duration of use).	10–18 to 28 weeks’ gestation
Borgen et al. (2019) [32] | Norway | 20	RCT	To investigate the effect of the Pregnant+ app on 2 h postpartum OGTT glucose levels in women with GDM.	**Pregnant+ |** Smartphone (operating system not specified)	Pregnant women with GDM; <33 weeks’ gestation | *n* = 238 (IG: 119; CG: 119). Three-month postpartum follow-up: IG: 115; CG: 123 | Age reported by age groups.	OGTT; clinical data; sociodemographic questionnaire; EQ-5D-5L; Fit for Delivery questionnaire; PPAQ; anthropometric assessment using BMI.	From GDM diagnosis to 3 months postpartum
Darvall et al. (2020) [33] | Australia | 18	RCT	To evaluate the feasibility of self-monitoring PA levels and the role of a smartphone-supported behavioural intervention in reducing the incidence of GWG.	**Fitbit |** Smartphone (iOS/Android)	Pregnant women; age >18 years; BMI ≥30 kg/m^2^; 12–16 weeks’ gestation | *n* = 30 (CG: 10; App IG: 10; AppCoach IG: 10). Final sample: *n* = 27 (CG: 8; App IG: 9; AppCoach IG: 10; 4 completed the intervention) | CG: 30.2 ± 5.3; App IG: 30.0 ± 5.0; AppCoach IG: 28.4 ± 5.8	Fitbit Zip; baseline BMI; Australian Physical Activity and Sedentary Behaviour Guidelines; Physical Activity Australia; Australian Dietary Guidelines.	25 weeks
Yew et al. (2021) [22] | Singapore | 22	RCT	To evaluate the effectiveness of the Habits-GDM app in preventing excessive gestational weight gain (GWG) and in improving glycaemic control and preventing maternal and perinatal complications associated with gestational diabetes mellitus (GDM).	**Habits-GDM** | Smartphone (operating system not specified)	Pregnant women with gestational diabetes mellitus (GDM); 12–16 weeks’ gestation | IG (*n* = 170): 31.7 ± 4.0; CG (*n* = 170): 32.2 ± 4.4	Oral glucose tolerance test (OGTT); Aina or Aina Mini glucometer; Habits-GDM app; Bluetooth-enabled weighing scale; Edinburgh Postnatal Depression Scale (EPDS); State-Trait Anxiety Inventory (STAI); clinical records.	From GDM diagnosis to delivery
Sandborg et al. (2021) [34] | Sweden | 23	RCT	To investigate the effectiveness of the HealthyMoms app intervention on gestational weight gain, body adiposity, dietary habits, moderate-to-vigorous physical activity (MVPA), blood glucose levels, and insulin resistance at 37 weeks’ gestation.	**HealthyMoms |** Smartphone (iOS/Android)	Pregnant women recruited at their first antenatal visit | IG (*n* = 152): 31.4 ± 4.3; CG (*n* = 153): 31.3 ± 3.8	HealthyMoms app; National Academy of Medicine recommendations; SATA software; Bod Pod (COSMED) for body weight and body composition assessment; Riksmaten FLEX and Swedish Healthy Eating Index for dietary assessment; ActiGraph wGT3X-BT accelerometer for physical activity assessment; COBAS 602 for glucose and insulin measurements; Homeostatic Model Assessment for Insulin Resistance (HOMA-IR).	6 months
Lim et al. (2021) [35] | Singapore | 21	RCT	To evaluate the effectiveness of an app-based intervention in achieving optimal postpartum weight among women with a recent history of gestational diabetes mellitus (GDM).	**nBuddy |** Smartphone (operating system not specified)	Postpartum women with a history of GDM | IG (*n* = 101): 32.6 ± 4.5; CG (*n* = 99): 32.4 ± 4.2. Final sample: IG: 96; CG: 93.	Seca 799 digital scale; oral glucose tolerance test (OGTT), HbA1c, and Homeostatic Model Assessment for Insulin Resistance (HOMA-IR); food diary; Health Education Impact Questionnaire (heiQ); RAND-12; Short-Form Health Survey; Self-Efficacy to Regulate Exercise; engagement assessed through manual backend data from the app dashboard and developer-provided usage statistics.	From delivery to 4 months postpartum
Bijlholt et al. (2021) [36] | Belgium | 19	RCT	To evaluate the effect of the Inter-ACT clinical trial on improving lifestyle indicators, including dietary intake, eating behaviour, physical activity, and sedentary time during the postpartum period.	**Inter-act |** Smartphone (operating system not specified)	Postpartum women with excessive GWG during pregnancy | Baseline: CG (*n* = 726): 31.2 ± 3.9; IG (*n* = 724): 31.4 ± 3.8. Final sample (6 months postpartum): CG = 390; IG = 435.	Validated Food Frequency Questionnaire (FFQ); Three-Factor Eating Questionnaire (TFEQ-R18); International Physical Activity Questionnaire (IPAQ); PA monitor (Withings Go); digital scale (Withings Body+).	Baseline: 6 weeks postpartum; end: 6 months postpartum; follow-up: 12 months postpartum
Smith et al. (2021) [37]| USA | 18	RCT	To evaluate the effect of a mobile meditation app on women’s well-being during the COVID-19 pandemic.	**Calm |** Smartphone (iOS/Android)	Pregnant women: IG 14–34 weeks; postsurgical women | IG (*n* = 50): 35.6 ± 9.9; CG (*n* = 51): 36.8 ± 12.6. Pregnant women (*n* = 60); other participants (*n* = 41).	Cohen’s Perceived Stress Scale (PSS); Zigmond and Snaith’s Hospital Anxiety and Depression Scale (HADS); Patient-Reported Outcomes Measurement Information System (PROMIS); final satisfaction questionnaire.	30 days
Gonzalez-Plaza et al. (2022) [38] | Spain | 17	RCT	Primary objective: To evaluate the effectiveness of a complex digital health intervention using a smart band and a mobile app on gestational weight gain and physical activity among women with obesity. Secondary objectives: To evaluate the impact of these interventions on maternal and perinatal outcomes and to assess the frequency of use, usability, and satisfaction with the mobile apps.	**Mi Fit |** Smartphone (iOS/Android)	Pregnant women; pre-pregnancy BMI ≥30 kg/m^2^; 12–18 weeks’ gestation | IG (*n* = 78): 32.4 ± 5.4; CG (*n* = 72): 33.4 ± 4.7	Mi Band 2 smart band; Mi Fit app; Hangouts app; International Physical Activity Questionnaire—Short Form (IPAQ-SF); Seca 704 scale; System Usability Scale (SUS); health counselling satisfaction questionnaires with Likert-scale items; clinical data.	From 12 to 18 weeks’ gestation to 35–37 weeks’ gestation
Nawabi et al. (2022) [39] | Germany | 16	Cluster RCT	To assess health literacy levels among pregnant women and to determine whether the GeMuKi intervention improved health literacy.	**GeMuKi |** Smartphone (iOS/Android)	Pregnant women; <12 weeks’ gestation | Total sample (general health literacy): *n* = 1010; total sample (specific health knowledge): *n* = 1016; IG: 31.3 ± 4.2; CG: 31.2 ± 4.4.	HLS-EU-16; Brief Health Literacy Screen (BHLS); baseline demographic data questionnaire; questionnaire for the objective assessment of pregnancy-related health literacy based on national recommendations.	From the beginning to the end of pregnancy
Chen et al. (2023) [28] | Taiwan | 18	RCT	To evaluate the effect of a nurse-led mHealth programme delivered through a mobile app on the management and prevention of excessive gestational weight gain (EGWG) among women with overweight and obesity.	**MyHealthyWeight (MHW) |** Smartphone (iOS/Android) + wearable Mi Band 5	Pregnant women; BMI ≥25 kg/m^2^; <17 weeks’ gestation | IG (*n* = 46): ≤35 years, *n* = 31; >35 years, *n* = 15; CG (*n* = 46): ≤35 years, *n* = 35; >35 years, *n* = 11. Final sample: *n* = 80 (IG: 37; CG: 43).	Clinical records; Pregnancy Physical Activity Questionnaire (PPAQ); diet and exercise self-efficacy questionnaire; Mi Band 5.	From <17 weeks’ gestation to 34–36 weeks’ gestation
Chen et al. (2024) [29]| Taiwan | 21	RCT	To analyse the effects of an mHealth intervention among women with overweight and obesity during pregnancy and up to 6 months postpartum.	**MyHealthyWeight (MHW) |** Smartphone (iOS/Android) + wearable Mi Band 5	Pregnant women; BMI ≥25 kg/m^2^; <17 weeks’ gestation | Baseline: *n* = 92 (IG: 46; CG: 46); follow-up (32 and 36 weeks’ gestation): IG: 37; CG: 43; final sample (6 months postpartum): *n* = 74 (IG: 36; CG: 38) | IG: ≤35 years: 70.3%; >35 years: 29.7%; CG: ≤35 years: 74.4%; >35 years: 25.6%.	Clinical records; baseline assessment questionnaire; PA data (steps/day measured using a wearable device).	20 weeks
Koeryaman et al. (2023) [40]| Indonesia | 21	RCT	To evaluate the effectiveness of the Sisfornutrimil app in achieving a balanced diet and healthy gestational weight gain (GWG), compared with women who did not use the app.	**Sisfornutrimil |** Smartphone (operating system not specified)	Pregnant women; 22–26 weeks’ gestation | IG (*n* = 56): 22.77 ± 2.37; CG (*n* = 56): 22.62 ± 2.04. Completed the study and final assessment: IG, *n* = 56; CG, *n* = 53.	Food records; Minimum Dietary Diversity for Women (MDD-W); Indonesian Recommended Dietary Allowances (RDA); weighing scale; independent-samples *t*-tests.	12 weeks
Hua et al. (2024) [41] | China | 20	RCT	To determine whether, and to what extent, self-efficacy for pelvic floor muscle training mediated by the UIW app improves the severity of postpartum urinary incontinence among pregnant women.	**Urinary Incontinence for Women (UIW) |** Smartphone (iOS/Android)	Pregnant women without urinary incontinence (UI) before pregnancy; 24–28 weeks’ gestation | IG (*n* = 51): 28.41 ± 3.48; CG (*n* = 52): 29.17 ± 2.96.	Baseline assessment questionnaire; Broome Pelvic Muscle Self-Efficacy Scale (BPMSES); International Consultation on Incontinence Questionnaire–Urinary Incontinence Short Form (ICIQ-UI-SF).	From 24 to 28 weeks’ gestation to 6 weeks postpartum
Zhou et al. (2025) [42]| Taiwan | 21	RCT	To determine whether an mHealth intervention based on the integrated Information–Motivation–Behavioural Skills (IMB) model improves psychological cognitions and behaviours related to gestational weight management.	**Pregnancy Assistant |** Smartphone (iOS/Android)	Pregnant women; <16 weeks’ gestation | T1: IG, *n* = 180; CG, *n* = 180; T2: IG, *n* = 168; CG, *n* = 170; T3: IG, *n* = 150; CG, *n* = 152 | IG: 29.31 ± 3.38; CG: 29.16 ± 3.49.	Questionnaires based on the Integrated Behavioural Model; gestational psychological cognition scales; gestational weight management behaviour scales.	From <16 weeks’ gestation to the end of pregnancy
Vadsaria et al. (2025) [43] | Pakistan | 21	RCT	To evaluate the effectiveness of a personalised mHealth coaching programme in improving dietary supplement intake during pregnancy.	**PurUmeed Aaghaz |** Smartphone (iOS/Android)	Pregnant women | IG: *n* = 153; CG: *n* = 153 | <25 years: IG, *n* = 22 (14.4%); CG, *n* = 26 (17.0%); 25–34 years: IG, *n* = 117 (76.5%); CG, *n* = 117 (76.5%); ≥35 years: IG, *n* = 14 (9.2%); CG, *n* = 10 (6.5%).	Structured questionnaires on regular dietary supplement intake; clinical data; Stata (version 14); data collection questionnaires.	24 weeks
Liang et al. (2025) [44]| China | 22	RCT	To evaluate the effects of a mobile app-mediated lifestyle intervention among postpartum women.	**Blessed Fruitful Pregnancy (BFP) |** (operating system not specified)	Postpartum women | IG: *n* = 75; CG: *n* = 75 | ≤24 years: IG, *n* = 5; CG, *n* = 4; 25–29 years: IG, *n* = 29; CG, *n* = 32; 30–34 years: IG, *n* = 20; CG, *n* = 20; 35–45 years: IG, *n* = 7; CG, *n* = 7.	Clinical records; questionnaires administered via the electronic system/app: Chinese version of the Edinburgh Postnatal Depression Scale (EPDS); Postpartum Fatigue Scale (PFS); Postpartum Sleep Quality Scale (PSQS).	Up to 42 days postpartum

Notes: RCT: randomized controlled trial; PA: physical activity; PE: physical exercise; GDM: gestational diabetes mellitus; App: application; OGTT: oral glucose tolerance test; IG: Intervention group; CG: control group; GWG: gestational weight gain; EGWG: excessive gestational weight gain; UIW app: Urinary Incontinence for Women; UI: urinary incontinence; iOS: iPhone operating system; MHW: MyHealthyWeight; BFP: Blessed Fruitful Pregnancy; BMI: body mass index; *n*: sample size.

**Table 2:** Results (authors, variables, intervention, results, conclusions).

Authors	Variables	Intervention	Results	Conclusions
Choi et al. [30]	PA (number of steps/day)	IG: mHealth app + Fitbit + feedback + messages + step goals; CG: Fitbit only.	Mean increase in the number of steps in the IG (not statistically significant); significant reduction in “lack of energy”; high initial app adherence, followed by a decline over time.	The intervention was feasible and acceptable; it did not demonstrate a significant effect on PA but showed potential for reducing barriers to PA.
Chan et al. [31]	P: Postpartum depression. S: Anxiety, stress, and quality of life.	IG: app + standard antenatal care; CG: standard antenatal care only.	Significant reduction in postpartum depression in the IG. No significant differences were observed for anxiety, stress, or quality of life.	The intervention was effective in reducing postpartum depression. The use of the app in conjunction with standard antenatal care may be effective in reducing postpartum depression at 4 weeks postpartum.
Kennelly et al. [26]	Incidence of GDM	IG: Initial face-to-face session (nutrition + exercise) + PEARS app + weekly motivational emails + initial consultation + two face-to-face follow-up consultations; CG: standard care.	The incidence of GDM did not differ between groups. The IG showed lower gestational weight gain, a lower dietary glycaemic load index, and increased exercise compared with the CG.	The behavioural intervention did not reduce the incidence of GDM but demonstrated benefits in lifestyle behaviours (↓ glycaemic load and ↑ exercise).
Ainscough et al. [27]	Diet, PA, behavioural change towards PA, app engagement	IG: standard antenatal care + education on diet and exercise + PEARS app + fortnightly emails + brief face-to-face contact at 28 and 34 weeks’ gestation; CG: standard antenatal care only.	IG: Improvements in dietary behaviours; PA increased in the IG, with the effects being more pronounced for diet than for PA.	The intervention was effective in reducing glycaemic load and glycaemic index and in improving nutritional intake; it also increased readiness to engage in PA. Greater app engagement appeared to be associated with greater benefits in dietary behaviour change than in PA.
Borgen et al. [32]	2 h plasma glucose following postpartum OGTT	IG: access to the Pregnant+ app + standard antenatal care; CG: standard antenatal care only.	No significant difference in 2 h plasma glucose following postpartum OGTT. No significant differences were observed in the assessed obstetric and neonatal outcomes.	The Pregnant+ app had no effect on 2 h plasma glucose following postpartum OGTT.
Darvall et al. [33]	P: Feasibility of self-monitoring. S: Effectiveness of pedometer feedback in increasing daily step count; benefit of investigator feedback compared with participant self-monitoring alone in reducing GWG.	CG: pedometer without access to step-count data; IG_app: pedometer + Fitbit Zip; IG_appcoach: pedometer + Fitbit Zip + behaviour change programme delivered by healthcare professionals.	Participant adherence was problematic (a high percentage of days with missing data + loss of four pedometers); data synchronisation with the smartphone was feasible in the IG_app and IG_appcoach groups. Mean daily step counts in all groups remained within the sedentary or low-activity categories.	There was no significant increase in PA.
Yew et al. [22]	P: Number of women with excessive GWG. S: Absolute gestational weight gain, glycaemic control, maternal and neonatal outcomes.	IG: standard antenatal care + Habits-GDM app, including 12 interactive lessons, self-monitoring of blood glucose and weight, automated feedback, and chat with healthcare professionals; CG: standard antenatal care only.	The intervention showed beneficial effects on the proportion of women with excessive GWG, absolute gestational weight gain, hypertensive disorders, and insulin use, although these differences were not statistically significant. Mean blood glucose levels were lower in the IG compared with the CG.	The app-based coaching programme did not significantly reduce the likelihood of excessive GWG compared with standard care. However, women in the IG showed better glycaemic control, which was associated with a reduction in adverse neonatal complications.
Sandborg et al. [34]	P: Gestational weight gain. S: Adiposity, dietary habits, moderate-to-vigorous PA, blood glucose, and insulin resistance.	IG: standard antenatal care + HealthyMoms app incorporating behaviour change techniques targeting dietary habits, PA, and healthy gestational weight gain; CG: standard antenatal care.	Women with pre-pregnancy overweight or obesity showed lower gestational weight gain in the IG compared with the CG. No statistically significant between-group differences were observed in adiposity, PA, blood glucose, or insulin resistance.	Although no overall effect on gestational weight gain was observed, the findings demonstrate the potential of the HealthyMoms app to promote healthy dietary behaviours and reduce gestational weight gain among women with overweight or obesity.
Lim et al. [35]	P: Achievement of “optimal” weight at 4 months postpartum. S: Absolute weight loss, 2 h OGTT, nutritional intake, quality of life assessed using questionnaires, and engagement with the app-based intervention.	IG: standard care + nBuddy initiated immediately after delivery, before hospital discharge; CG: standard care + clinical follow-up at 6 weeks postpartum + OGTT + dietary counselling.	P: 40% of women in the IG vs. 32% in the CG achieved optimal weight at 4 months postpartum (marginally significant difference). S: Marginally significant differences in anthropometric measurements and OGTT outcomes; significant reduction in total caloric intake in the IG at 6 weeks and 4 months; marginally significant increase in the number of steps in the IG between 6 weeks and 4 months; at 4 months, women in the IG reported greater confidence and ability to maintain exercise compared with the CG, as well as higher levels of health-oriented behaviours. Engagement: 60.8% at 4 months.	The app enabled substantially greater engagement with postpartum lifestyle behaviours than previous interventions. Although it did not significantly increase the number of women achieving “optimal weight” at 4 months postpartum, it appeared to improve health behaviours while maintaining high levels of user engagement.
Bijlholt et al. [36]	Dietary intake; eating behaviour; PA; time spent in sedentary behaviour; mental well-being.	IG: four coaching sessions at 6, 8, and 12 weeks and 6 months postpartum + Inter-ACT app, providing motivational messages and informative tips and allowing participants to monitor their goals; connected to a PA monitor and weighing scale for self-monitoring of PA and weight. CG: standard postpartum care.	The Inter-ACT app intervention was effective in reducing uncontrolled eating and energy intake. No significant effects were observed on emotional eating, PA, or time spent in sedentary behaviour.	The Inter-ACT intervention was effective in improving dietary outcomes but not PA or time spent in sedentary behaviour. The observed improvements were not maintained at the 6-month follow-up.
Smith et al. [37]	P: Perceived stress. S: Depression, anxiety, sleep disturbances, and satisfaction with the app.	IG: standard care + Calm app; CG: standard care.	Obstetric participants (*n* = 60) reported significantly lower stress than the CG at day 30 and significantly lower depression and sleep disturbances at day 14. At day 30, depression, sleep disturbances, and anxiety were lower in the obstetric IG, although the differences were not statistically significant. More than 80% of participants in the IG reported high satisfaction with the Calm app.	The use of a mindfulness app for 30 days was associated with significant reductions in stress, depression, anxiety, and sleep disturbances compared with standard care during the COVID-19 pandemic.
Gonzalez-Plaza et al. [38]	P: Gestational weight gain; total PA and time spent in sedentary behaviour. S: Incidence of maternal complications; incidence of labour induction, mode of delivery, and unplanned caesarean section; incidence of perinatal complications.	IG: standard care + combined digital intervention based on Social Cognitive Theory, using a smart band connected to the Mi Fit app for PA monitoring + Hangouts app for communication and personalised health information; CG: standard care.	P: Median gestational weight gain was 7 kg in the IG compared with 9.3 kg in the CG; mean GWG/week was higher in the CG. The proportion of women with adequate GWG was 35% in the IG vs. 28% in the CG; GWG below recommendations: 30% vs. 17%; GWG above recommendations: 35% vs. 55%, respectively. Between 35 and 37 weeks, women in the IG showed higher mean total PA levels and lower mean sedentary time than those in the CG. S: No between-group differences were observed in the incidence of maternal or perinatal outcomes. In the IG, 75% rated usability as excellent. Mean app satisfaction score was 4.8/5 (SD 0.6).	The intervention was associated with lower GWG and increased PA during pregnancy among women with obesity. No differences in maternal or perinatal complications were observed between the two study groups. The usability of the app combined with the smart band was rated as excellent, and overall satisfaction with the app was very high.
Nawabi et al. [39]	P: Prevention of excessive gestational weight gain. S: Improvement in maternal and neonatal health outcomes; improvement in health literacy.	IG: additional lifestyle counselling tailored to pregnant women’s needs, focusing on nutrition and PA + GeMuKi app providing pregnancy-related health information; CG: usual counselling.	No significant effect of the GeMuKi intervention on general health literacy was observed.	The GeMuKi intervention did not improve general health literacy but was effective in improving specific knowledge related to lifestyle during pregnancy.
Chen et al. [28]	P: Incidence of excessive gestational weight gain; gestational weight gain trajectory and proportion of participants with excessive weight gain. S: Factors associated with weight gain throughout pregnancy among women with obesity; behavioural changes related to weight, dietary intake, and PA.	IG: standard care + MyHealthyWeight app + wearable PA device; CG: standard care.	P: Although fewer women in the IG experienced excessive total GWG compared with the CG, the between-group difference was not statistically significant. Mean body weight and mean GWG also did not differ significantly between groups. S: Among pregnant women with obesity, pre-pregnancy exercise habits, greater perceived dietary self-efficacy, and higher PA levels were associated with lower GWG. In the IG, women with overweight or obesity increased their step count and maintained their mean daily step count throughout pregnancy.	The intervention demonstrated beneficial effects, including lower GWG and increased dietary self-efficacy for achieving adequate GWG, particularly among women with obesity. Women with higher BMI benefited more from the intervention. Among women with obesity, pre-pregnancy exercise habits, greater perceived dietary self-efficacy, and higher PA levels were associated with lower GWG. Among women with overweight or obesity, the intervention also increased step counts and supported the maintenance of PA levels.
Chen et al. [29]	P: Gestational weight gain. S: Maternal weight; weight retention at 6 months postpartum; neonatal birth weight.	IG: standard antenatal care + one-to-one counselling via the MyHealthyWeight app + Mi Smart Band 5; CG: standard antenatal care.	The IG showed a trend towards lower mean body weight than the CG at the final assessment before delivery and at 6 months postpartum, although the differences were not statistically significant. Mean weight in the IG tended to remain lower than in the CG throughout pregnancy and the postpartum period. Women with obesity in the IG showed significantly lower weight before delivery compared with those in the CG. Mean neonatal birth weight was significantly lower in the IG.	The intervention supported effective management of gestational weight gain and pre-delivery body weight among women with obesity, although this effect was not maintained at 6 months postpartum.
Koeryaman et al. [40]	Dietary diversity; macro- and micronutrient intake; gestational weight gain.	Pregnant women were instructed to consume at least five food groups and follow the recommended average portion sizes for 12 weeks. Both groups recorded their dietary intake for 3 days/week. Dietary diversity, macro- and micronutrient intake, and gestational weight gain were assessed.	Mean dietary diversity score was significantly higher in the IG. Macro- and micronutrient intake was significantly more closely aligned with recommendations in the IG compared with the CG, with higher vitamin and mineral intake. Gestational weight gain in the IG was within healthy weight-gain parameters, whereas mean energy intake in the CG did not meet the recommendations.	Dietary intake recording through the app was significantly more effective than the traditional recording method in improving dietary diversity, achieving adequate energy and nutrient intake, and promoting healthy gestational weight gain.
Hua et al. [41]	P: Severity of postpartum urinary incontinence. S: Self-efficacy for pelvic floor muscle training.	IG: UIW app + verbal instructions on pelvic floor muscle training (education and guidance provided by obstetricians); CG: verbal instructions only.	The UIW app-based intervention increased self-efficacy for pelvic floor muscle training. The intervention may positively influence urinary incontinence management in pregnant women by increasing their confidence and motivation to perform pelvic floor muscle exercises.	The UIW app-based intervention increased self-efficacy for pelvic floor muscle training, and this increase was associated with an improvement in the severity of postpartum urinary incontinence. The UIW app demonstrated clinically relevant effectiveness.
Zhou et al. [42]	P: Cognitions related to gestational weight management. S: Gestational weight-management behaviours (healthy eating and PA); gestational weight gain.	IG: mobile app + fortnightly sessions delivered by a specialist (including nutrition and PE); CG: standard antenatal care, with information provided through the app.	The IG showed significantly higher scores in the domains of information, perceived vulnerability, and PE management in the second trimester, as well as significantly higher scores in perceived vulnerability and dietary management in the third trimester, compared with the CG.	The intervention was effective in improving psychological cognitions. The app played an important role as an effective means of delivering information and facilitating social interaction among women.
Vadsaria et al. [43]	P: Adherence to micronutrient supplementation. S: Knowledge of supplementation and related behaviours.	IG: personalised coaching through the PurUmeed Aaghaz app, with push notifications three times/week and tailored recommendations over 24 weeks; CG: standard counselling.	A relative improvement of 30% was observed in adequate intake of folic acid, iron, calcium, and vitamin D. The proportion of iron supplement users was higher in the IG, and calcium supplementation use was significantly greater in the IG. Vitamin D supplementation use was similar between groups. Serum ferritin, calcium, and vitamin D levels did not show improvements at the end of the study in either group. Ferritin and calcium deficiencies increased in the CG. Maternal and neonatal outcomes did not differ significantly between groups. Although the prevalence of gestational diabetes was higher in the CG than in the IG, this difference was not statistically significant.	The personalised mHealth intervention improved the use of the four assessed micronutrient supplements. No improvements were observed in ferritin, calcium, and vitamin D levels over time. Iron and calcium deficiencies worsened in the CG. mHealth-based counselling may contribute to improving micronutrient status during pregnancy. The positive role of the mHealth intervention in improving daily iron supplementation was evident, although the differences were not statistically significant.
Liang et al. [44]	P: Prevention of postpartum symptoms (constipation, prolonged lochia, and pelvic floor dysfunction). S: Sleep quality, postpartum fatigue, and depressive symptoms.	IG: standard care + app. Baseline assessments were conducted between the 2nd and 5th postpartum days, with outcomes collected at 42 days postpartum; CG: standard postpartum care.	The IG showed significant improvements in sleep quality, fatigue, and depressive symptoms compared with the CG. No statistically significant differences were observed between groups regarding the incidence of pelvic floor dysfunction.	The intervention was effective in preventing postpartum constipation and prolonged lochia, as well as improving depressive symptoms, fatigue, and sleep quality.

Notes: P: principal; S: secondary; PA: physical activity; GDM: gestational diabetes mellitus; MET: metabolic equivalent task; App: application; PE: physical exercise; OGTT: oral glucose tolerance test; ↑: increase; ↓: significant decrease; IG: intervention group; CG: control group; GWG: gestational weight gain; vs.: versus; uMARS: User Mobile App Rating Scale; UIW app: Urinary Incontinence for Women.

**Table 3:** Functionalities, characteristics and effectiveness of each study with mHealth apps.

*App*	Functionalities											Characteristics					Effectiveness
	H.E.	S.M.	FB	Pers	Diet	P.A.	Stress	Sleep	I.H.P	S.F.	A.I.	I.Q.	Usab.	S&P	C.A.	Access.	
**MoTHER**	**Yes**	Yes	Yes	Yes	Yes	Yes	No	No	Partial	No	No	Yes	Partial	Partial	Yes	Partial	Yes
**iParent**	**Yes**	No	Partial	No	Yes	No	Yes	No	Yes	Partial	No	Yes	Partial	No	Yes	Yes	Yes
**PEARS**	**Yes**	No	Partial	Yes	Yes	Yes	No	No	Partial	No	No	Yes	Partial	No	Yes	Yes	Yes
**PEARS**	**Yes**	No	Partial	Yes	Yes	Yes	No	No	Partial	No	No	Yes	Yes	Partial	Yes	Yes	Yes
**Pregnant+**	Yes	Yes	Yes	Yes	Yes	Yes	No	No	Partial	No	No	Yes	Yes	Partial	Yes	Yes	No
**Fitbit**	**Yes**	Yes	Partial	Yes	No	Yes	No	No	Yes	No	No	Yes	Yes	Partial	Yes	Yes	No
**Habits-GDM**	**Yes**	Yes	Yes	Yes	Yes	Yes	Partial	No	Yes	No	No	Yes	Partial	Yes	Yes	Partial	Yes
**HealthyMoms**	**Yes**	Yes	Yes	Yes	Yes	Yes	No	No	No	No	No	Yes	Yes	Partial	Yes	Yes	Yes
**nBuddy**	**Yes**	Yes	Yes	Yes	Yes	Yes	Partial	No	Yes	No	No	Yes	Yes	Partial	Yes	Partial	Yes
**Inter-act**	**Yes**	Yes	Yes	Yes	Yes	Yes	Yes	No	Yes	Partial	No	Yes	Yes	Partial	Yes	Partial	Yes
**Calm**	**Yes**	No	No	Partial	No	No	Yes	Yes	No	No	No	Yes	Yes	Partial	No	Yes	Yes
**Mi Fit**	**Yes**	Yes	Yes	Yes	Yes	Yes	No	No	Yes	No	No	Yes	Yes	Yes	Yes	Yes	Yes
**GeMuKi**	**Yes**	Partial	Yes	Yes	Yes	Yes	No	No	Yes	No	No	Yes	Partial	Yes	Yes	Partial	Yes
**MHW**	**Yes**	Yes	Yes	Yes	Yes	Yes	No	No	Yes	No	No	Yes	Yes	Partial	Yes	Partial	Yes
**MHW**	**Yes**	Yes	Yes	Yes	Yes	Yes	No	No	Yes	No	No	Yes	Partial	Partial	Yes	Partial	Yes
**Sisfornutrimil**	**Yes**	Yes	Yes	Partial	Yes	No	No	No	Partial	No	No	Yes	Partial	Partial	Yes	Yes	Yes
**UIW**	**Yes**	Partial	Yes	Partial	No	Yes	No	No	Yes	No	No	Yes	Partial	Partial	Yes	Partial	Yes
**Pregnancy Assistant**	**Yes**	Yes	Yes	Yes	Yes	Yes	No	No	Partial	Partial	No	Yes	Partial	Partial	Yes	Partial	Yes
**PurUmeed Aaghaz**	**Yes**	Yes	Yes	Yes	Yes	No	No	No	Yes	No	No	Yes	Partial	Yes	Yes	Yes	Yes
**BFP**	**Yes**	Yes	Yes	Yes	Yes	Yes	Partial	Partial	Yes	Partial	No	Yes	Partial	Partial	Yes	Partial	Yes

Notes: H.E.: health education; S.M.: self-monitoring; FB: feedback; Pers: personalisation; P.A.: physical activity. I.H.P.: interaction with healthcare professionals; S.F.: social features; AI: artificial intelligence; I.Q.: information quality; Usab: usability; S&P: security and privacy; C.A.: clinical appropriateness; Access.: accessibility; MHW: MyHealthyWeight; UIW: Urinary Incontinence for Women app; BFP: Blessed Fruitful Pregnancy app.

Regarding the geographical distribution of the interventions, eight studies were conducted in Europe (Ireland, Germany, Spain, Norway, Sweden, and Belgium), three studies were conducted in the United States, China, and Taiwan, two studies were conducted in Singapore, and the remaining studies were carried out in Indonesia, Pakistan, and Australia (see Table 1 and Table 2).

### 3.3. Study Quality and Analysis of Study Results

Regarding the quality assessment according to the Downs and Black checklist, scores ranged from 16 to 23 points (M = 20.00 ± 1.45 points). Nine articles were classified as having fair quality, while eleven were classified as having good quality (see Table 1).

The results are described in Table 1, Table 2 and Table 3.

#### 3.3.1. Effectiveness of the Applications

Of the eighteen applications analysed, two showed no evidence of effectiveness within the scope of the intervention in which they were used, as the primary and/or secondary outcomes defined in the studies were not achieved. These were Pregnant+ [32] and Fitbit [33] (Table 1, Table 2 and Table 3).

In the study by Borgen et al. [32], using the Pregnant+ app, no effect was observed on 2 h plasma glucose levels after the oral glucose tolerance test (OGTT) in the postpartum period, nor were differences observed regarding obstetric and neonatal outcomes. In the study by Darvall et al. [33], whose intervention included the Fitbit app, no increase in physical activity was observed (Table 1 and Table 2).

Among the remaining applications, iParent [31], Habits-GDM [22], HealthyMoms [34], Mi Fit [38], MyHealthyWeight [29], and Urinary Incontinence for Women [41] demonstrated effectiveness regarding clinical outcomes; MoTHER [30], nBuddy [35], Inter-act [36], GeMuKi [39], and Pregnancy Assistant [42] demonstrated effectiveness regarding behavioural outcomes; while Calm [37], MyHealthyWeight [28], Sisfornutrimil [40], PurUmeed Aaghaz [43], and Blessed Fruitful Pregnancy [44] demonstrated effectiveness regarding both clinical and behavioural outcomes. These findings reinforce the importance of healthy lifestyle behaviours associated with the adoption of health-promoting behaviours throughout the perinatal period.

#### 3.3.2. Characteristics of the Applications

For data extraction, the following app characteristics were considered: information quality, usability, security and privacy, clinical appropriateness, and accessibility (Table 3). Among all characteristics analysed, information quality was the only characteristic present in all apps classified as effective, followed by clinical appropriateness, usability, accessibility, and, lastly, security and privacy.

#### 3.3.3. Functionalities of the Applications

The app functionalities, also described in the background section of this study, included 11 domains: health education, self-monitoring, feedback, personalisation, interaction with healthcare professionals, social features, and artificial intelligence (AI) integration, as well as healthy lifestyle behaviours related to diet, physical activity, stress/mental health, and sleep (Table 3).

The functionality present in all applications was health education, followed by personalisation, self-monitoring, feedback, and interaction with healthcare professionals. Regarding functionalities directly associated with healthy lifestyle behaviours, diet was the domain included in the highest number of applications, followed by physical activity, whereas stress and sleep were addressed in a substantially smaller number of applications (Table 3).

## 4. Discussion

The primary aim of this systematic review was to analyse the effectiveness of mobile applications in promoting healthy lifestyle behaviours (HLB) during pregnancy and the postpartum period, while the secondary aim was to identify the characteristics and functionalities required or desirable in apps targeting the perinatal period.

The twenty included studies demonstrated fair to good methodological quality, with scores ranging from 16 to 23 out of a possible 28 points according to the Downs and Black checklist (Figure 1).

In all studies, random allocation of the intervention was not fully achieved, either among participants or healthcare professionals. In addition to this criterion, the lack of population representativeness and the absence of blinding of participants and assessors were items from the checklist that were not fulfilled in most studies.

The studies included in this review were published between 2016 and 2025, with fourteen published within the last five years, reflecting the increasing growth and relevance of mHealth applications in healthcare.

Figure 2 shows an infographic of this systematic review.

### 4.1. Effectiveness of the Applications

Of the eighteen apps analysed, two were considered to have no demonstrated effectiveness within the context of the RCT intervention in which they were implemented: Pregnant+ [32] and Fitbit [33].

Rather than indicating that mHealth applications are intrinsically ineffective, these findings highlight the importance of considering the characteristics of the intervention context, target outcomes, adherence, sample characteristics, and implementation when interpreting effectiveness. For example, the Pregnant+ intervention targeted a specific clinical outcome related to glucose regulation in women with GDM, while the Fitbit intervention was conducted with a small sample and was affected by missing data and implementation difficulties.

The included interventions should be interpreted as complex app-based interventions rather than as isolated mobile application interventions. In several studies, the application was integrated with wearable devices, behavioural support, counselling, healthcare-professional involvement, or other digital components. Therefore, the observed effects cannot necessarily be attributed exclusively to the mobile application. Rather, the findings reflect the effectiveness of the intervention packages in which the mobile application constituted an integral component.

The predominance of behavioural and lifestyle-related effects observed across the included studies may also be relevant. Most interventions with clinical primary outcomes incorporated behavioural secondary outcomes, suggesting that mHealth applications may initially influence behaviours such as dietary intake, physical activity, or self-management before measurable changes in clinical outcomes become evident. This supports the consideration of both behavioural and clinical outcomes when evaluating interventions aimed at promoting healthy lifestyles during the perinatal period.

### 4.2. Characteristics of the Applications

The characteristic ‘information quality’ was present in all applications classified as effective, followed by clinical appropriateness, usability, accessibility, and security and privacy. However, these findings should be interpreted descriptively, as the review did not formally test associations between app characteristics or functionalities and intervention effectiveness. This finding is consistent with a qualitative study analysing feedback from women using pregnancy-related mHealth apps, in which participants reported that information quality and its appropriateness to the perinatal stage significantly influenced their satisfaction with these tools (Table 3) [45]. These findings are also aligned with WHO recommendations regarding digital health interventions, which highlight the importance of safety, accessibility, and information quality [7].

However, it should be noted that, among the apps analysed in this systematic review, two did not meet the security and privacy criterion [30,32], and 14 only partially met this criterion [26,27,28,29,33,34,35,36,37,38,39,40,41,42,43,44] (Table 3). Reproductive health apps targeting pregnancy and postpartum care often include sensitive personal and clinical information, and gaps in privacy policies are frequently identified, raising additional concerns regarding user safety and data protection.

A study evaluating the quality of pregnancy and postpartum apps concluded that, although many achieved high scores in technical domains, they presented weak security and privacy policies, particularly regarding data collection and sharing with third parties [10]. Therefore, based on current evidence, security and privacy should be prioritised in the development of future perinatal health apps.

Accessibility was considered partial in ten apps, mainly because they were not free of charge or because this characteristic was not described in the study intervention (Table 3). WHO (2019) emphasises that access to digital health technologies should be equitable; therefore, free access to mHealth apps should be considered an important element in promoting the adoption and use of these tools while reducing inequalities [7].

Usability was considered partial in eleven of the eighteen analysed apps [22,28,29,30,31,39,40,41,42,43,44].

This finding suggests that usability remains insufficiently addressed across perinatal mHealth interventions, particularly when user experience is not systematically assessed using validated instruments. In interventions using PEARS [27], nBuddy [35], and Inter-act [36], validated instruments were not used to assess user experience. Several studies focused primarily on clinical outcomes, such as weight, glucose levels, and symptoms of specific clinical conditions, rather than user experience and ease of use, as observed in Fitbit [33], Pregnant+ [32], and MoTHER [30].

In studies involving apps such as MyHealthyWeight (MHW) [28], Urinary Incontinence for Women (UIW) [41], and nBuddy [35], these applications were research prototypes developed specifically for the intervention. The study involving Inter-act [36] reported early dropout and limited use of functionalities. Together, these observations suggest that usability should be considered not only as a technical characteristic of the application but also in relation to sustained engagement and continued use throughout the intervention.

The Mi Fit app [38] incorporated all five assessed characteristics: information quality based on physical activity practice recommendations; usability through a simple interface; security and privacy associated with account creation and compliance with privacy policies; accessibility through free availability; and clinical appropriateness for the perinatal period, particularly through objective monitoring of physical activity parameters using a wearable device.

Therefore, future mHealth apps targeting pregnancy and postpartum care should consider users’ security, privacy, accessibility, and usability when designing interventions intended to support effective health promotion.

### 4.3. Functionalities of the Applications

Among the 11 functionalities considered in this study, health education was the only functionality present in all analysed apps. Health education during pregnancy and the postpartum period is considered essential for promoting healthy lifestyles and supporting informed decision-making, with the aim of contributing to improved maternal, foetal, and neonatal well-being [46].

Personalisation, self-monitoring, and feedback were simultaneously present in eleven different apps [22,28,29,31,32,35,36,37,39,42,43].

A study conducted by Yu et al. compared the functionalities of pregnancy and postpartum mHealth apps available in China and the United States, concluding that the analysed apps clearly prioritised health education and self-monitoring functionalities, particularly regarding parameters such as weight, fluid intake, and exercise practice [47]. Raab et al. considered that feedback generated from monitored lifestyle data and goals increases motivation and engagement among women using apps during the perinatal period [48]. Furthermore, Jones et al. identified personalisation as an essential functionality in pregnancy and postpartum mHealth apps, as the individualisation of content and recommendations, combined with feedback based on self-monitoring, may increase usability and facilitate the adoption of healthy behaviours among pregnant and postpartum women [49].

Interaction with healthcare professionals was a functionality included in eleven apps analysed in this study [28,29,30,32,33,34,36,38,42,43,44]. According to Liblub et al., the quality of information supported by scientific evidence and healthcare professionals is fundamental in mHealth apps, representing a frequent gap in pregnancy and postpartum digital health tools [50]. Similarly, Alves et al. emphasised the importance of involving healthcare professionals in the design, development, evaluation, and validation of mHealth applications to ensure that the information provided is evidence-based, up to date, and responsive to women’s healthcare needs [12]. Although more than half of the apps analysed in this systematic review included interaction with healthcare professionals, this remains an area requiring further improvement in future perinatal health apps, in order to enhance women’s safety and confidence during their use.

Only four apps partially incorporated social features through interaction with other users, and none described the use of artificial intelligence (AI) based on algorithms or machine learning systems.

Regarding functionalities related to healthy lifestyle behaviours (HLB), diet and physical activity were included in fifteen [22,26,27,28,29,30,31,32,34,35,36,38,39,40,42,43,44] and fourteen apps, respectively [22,26,27,28,29,30,32,33,34,35,36,38,39,41,42,44], highlighting their relevance as primary and/or secondary outcomes in a large proportion of the analysed apps.

Mental health-related stress management was included in only three apps [31,36,37], and was partially addressed in three additional apps [22,35,44]. Sleep was identified in only two apps [37,44], with only partial inclusion in the latter.

The relatively limited incorporation of stress management and sleep-related functionalities is noteworthy, given the relevance of psychological well-being and sleep during the perinatal period. This suggests that future applications aiming to promote comprehensive healthy lifestyles should move beyond the more commonly addressed domains of diet and physical activity and incorporate a broader range of behavioural targets.

Furthermore, interaction with healthcare professionals was limited, consisting mainly of predefined content and guidance without direct communication, which may have contributed to the observed results. Therefore, the effectiveness of mHealth apps may depend not only on the number of characteristics and functionalities included, but also on their quality and their ability to promote sustained engagement among pregnant women and mothers.

However, integrating multiple functionalities and characteristics within an app may be advantageous when these are implemented with simplicity and personalisation, avoiding user overload and/or loss of interest. This aspect is particularly relevant during periods characterised by multiple physical, psychological, and emotional changes, requiring continuous adaptation to new conditions and demands [51].

Based on current evidence, the development of a future app aimed at promoting HLB is intended, including physical exercise, diet, improved sleep quality, and stress regulation, while also facilitating multidisciplinary intervention among healthcare and exercise professionals involved in the care of pregnant and postpartum women.

### 4.4. Limitations

The literature search was conducted in three electronic databases (PubMed, Web of Science, and EBSCO), selected to provide complementary coverage of biomedical, multidisciplinary, and health-related literature. Nevertheless, the omission of other databases may have resulted in potentially eligible studies not being identified and should therefore be considered when interpreting the findings.

The search strategy may also have limited the retrieval of potentially eligible interventions focused exclusively on health education when these studies did not include terms related to exercise, physical activity, lifestyle, nutrition, sleep, or stress in the searchable record.

The quality assessment of the included studies using the Downs and Black checklist [24] resulted in nine articles being classified as having fair quality and 11 as having good quality. It should be highlighted that, regarding the external validity item, all analysed studies received a score of zero, as it was not possible for participants to be unaware of the use of the app. Additionally, it was not possible to ensure that the professionals responsible for delivering the interventions were blinded to the outcomes obtained [24].

Other methodological limitations should also be acknowledged, particularly the heterogeneity of assessment instruments, variability in sample characteristics, and the short duration of interventions. Furthermore, there was limited and unclear reporting of app functionalities and characteristics, which may compromise the robustness of the evidence regarding effectiveness and, consequently, the generalisability of the findings.

These limitations affect study comparability and the generalisation of results and should be considered in future research. They reinforce the need for greater methodological standardisation and more robust approaches to evaluate the effectiveness of mHealth interventions during pregnancy and the postpartum period.

### 4.5. Implications for Future Research

Considering the limitations identified in the analysed studies and the recommendations for the development of mHealth applications, future research should focus on:

- The development of an mHealth app (Active Pregnancy App) based on the functionalities and characteristics identified in this review, to be evaluated through an intervention study with a representative sample;
- The assessment of the effectiveness of app-based interventions promoting HLB during the perinatal period over the medium and long term, through follow-up assessments after the intervention period;
- The evaluation of the implementation of an exercise programme during pregnancy and the postpartum period, and its impact on maternal health outcomes throughout these stages.

## 5. Conclusions

The promotion of healthy lifestyle behaviours during pregnancy and the postpartum period plays a crucial role in improving maternal, foetal, and neonatal health. Currently, there has been a growing number of mHealth apps targeting pregnancy and postpartum care, increasingly used by women as complementary sources of information alongside conventional antenatal and postnatal healthcare.

The findings of this systematic review demonstrate that, although increasing evidence supports the potential of mobile health applications, the interventions implemented across the twenty included studies using eighteen pregnancy- and postpartum-specific apps were complex and heterogeneous. While these interventions may contribute to improvements in behavioural outcomes related to healthy lifestyle behaviours, evidence regarding their effectiveness in improving clinical outcomes remains less consistent.

The effectiveness of apps promoting HLB during the perinatal period appears to be associated with characteristics such as information quality and clinical appropriateness. However, security and privacy, usability, and accessibility demonstrated lower consistency across studies. Therefore, these characteristics should be incorporated into future mHealth applications to improve their effectiveness.

Regarding app functionalities, health education was present in all apps demonstrating effectiveness, whereas self-monitoring, feedback, personalisation, and interaction with healthcare professionals were included in fewer apps and were not always clearly described.

In conclusion, the development of mHealth apps represents an opportunity to integrate these dimensions into an evidence-based and comprehensive digital health tool, capable of addressing women’s needs throughout pregnancy and the postpartum period, promoting the adoption of healthy lifestyle behaviours and improving maternal, foetal, and neonatal outcomes during these stages.

## Figures and Tables

**Figure 1:**
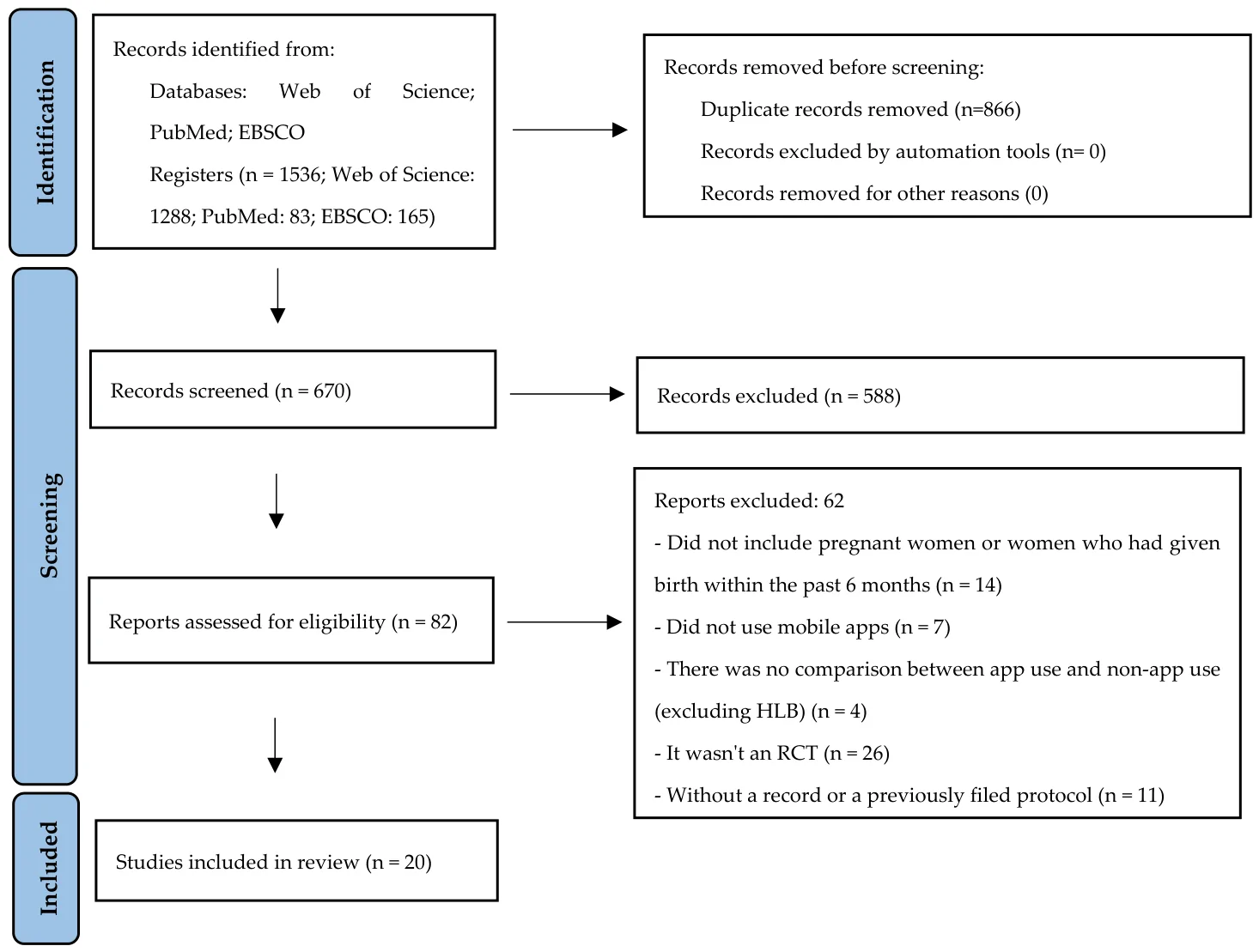
PRISMA flow diagram of the study selection process.

**Figure 2:**
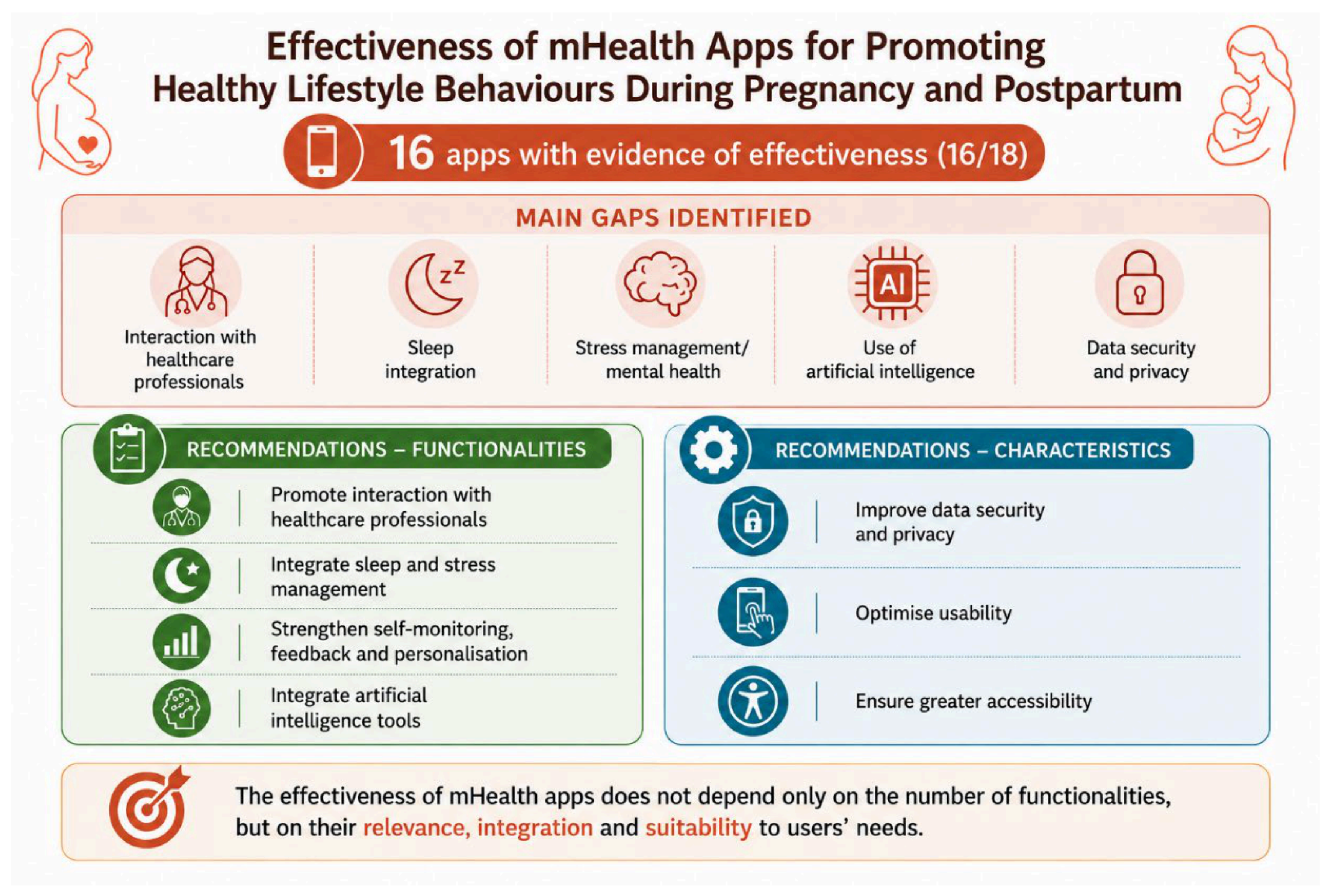
Systematic review infographic (developed by the authors with the assistance of ChatGPT, [GPT-5.3 Instant], OpenAI; last modified September 2026).

## Data Availability

No new data were created or analysed in this study.
